# Nanosecond formation of diamond and lonsdaleite by shock compression of graphite

**DOI:** 10.1038/ncomms10970

**Published:** 2016-03-14

**Authors:** D. Kraus, A. Ravasio, M. Gauthier, D. O. Gericke, J. Vorberger, S. Frydrych, J. Helfrich, L. B. Fletcher, G. Schaumann, B. Nagler, B. Barbrel, B. Bachmann, E. J. Gamboa, S. Göde, E. Granados, G. Gregori, H. J. Lee, P. Neumayer, W. Schumaker, T. Döppner, R. W. Falcone, S. H. Glenzer, M. Roth

**Affiliations:** 1 Department of Physics, University of California, Berkeley, California 94720, USA; 2 SLAC National Accelerator Laboratory, Menlo Park, California 94025, USA; 3 Centre for Fusion, Space and Astrophysics, Department of Physics, University of Warwick, Coventry CV4 7AL, UK; 4 Max-Planck-Institut für Physik Komplexer Systeme, Nöthnitzer Strasse 38, 01187 Dresden, Germany; 5 Institute of Radiation Physics, Helmholtz-Zentrum Dresden-Rossendorf, Bautzner Landstrasse 400, 01328 Dresden, Germany; 6 Institut für Kernphysik, Technische Universität Darmstadt, Schlossgartenstrasse 9, 64289 Darmstadt, Germany; 7 Lawrence Livermore National Laboratory, Livermore, California 94550, USA; 8 Department of Physics, University of Oxford, Parks Road, Oxford OX1 3PU, UK; 9 GSI Helmholtzzentrum für Schwerionenforschung GmbH, Planckstrasse 1, 64291 Darmstadt, Germany

## Abstract

The shock-induced transition from graphite to diamond has been of great scientific and technological interest since the discovery of microscopic diamonds in remnants of explosively driven graphite. Furthermore, shock synthesis of diamond and lonsdaleite, a speculative hexagonal carbon polymorph with unique hardness, is expected to happen during violent meteor impacts. Here, we show unprecedented *in situ* X-ray diffraction measurements of diamond formation on nanosecond timescales by shock compression of pyrolytic as well as polycrystalline graphite to pressures from 19 GPa up to 228 GPa. While we observe the transition to diamond starting at 50 GPa for both pyrolytic and polycrystalline graphite, we also record the direct formation of lonsdaleite above 170 GPa for pyrolytic samples only. Our experiment provides new insights into the processes of the shock-induced transition from graphite to diamond and uniquely resolves the dynamics that explain the main natural occurrence of the lonsdaleite crystal structure being close to meteor impact sites.

## Abstract (web summary)

Shock synthesis of diamond and even harder carbon polymorphs from graphite is of great interest for science and technology. Here, the authors present unprecedented *in situ* measurements of the structural changes, showing ultrafast formation of diamond and, at higher pressures, evidence for a pure lonsdaleite structure.

## Graphical abstract

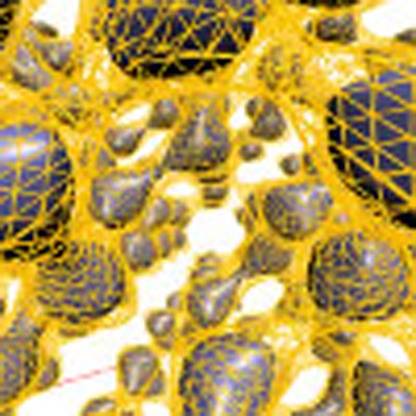

The formation of diamond after applying pressure and heat on graphite is highly relevant to the artificial synthesis of diamond^1^,^2^,^3^,^4^, and also for a general understanding of high pressure phase transitions in environments comparable to meteor impacts or planetary interiors^5^,^6^. In 1967, a hexagonal form of diamond^7^, later called lonsdaleite^8^, in its pure form supposedly harder than diamond^9^, was identified for the first time inside fragments of the Canyon Diablo meteorite, the asteroid which created the Barringer Crater with an impact velocity of ∼10 km s^−1^ generating pressures exceeding 200 GPa (ref. 5). Since then, occurrences of lonsdaleite and also nanometer-sized diamonds have been suggested to serve as marker for meteor impacts all over the world, for example, the Tunguska explosion in the early twentieth century^10^, the Ries crater^11^, the Younger Dryas event^12^ or the impact which has been correlated to the extinction of the dinosaurs^13^. However, due to difficulties in creating a pure lonsdaleite phase under static compression conditions, serious doubts on the overall existence of lonsdaleite were published recently^2^,^14^. Yet, static compression is not well suited to mimic the fast processes during impact events.

Dynamic shock compression experiments, driven by explosives^15^,^16^, gas guns^4^,^17^ or pulsed high-energy lasers^18^, are much closer to such conditions despite the smaller dimensions and thus shorter timescales (nanoseconds to microseconds) compared with meteor impacts (seconds). For this reason, laboratory shock experiments are limited to detect phase transitions happening on corresponding timescales and cannot explore some complex structural changes, which are thought to require nucleation times up to seconds^19^. The shock-induced transition from graphite to diamond is thought to be a fast martensitic transition^4^, but up to now, shock compression experiments on graphite have lacked a direct *in situ* measurement of the microscopic crystal structure. In fact, all conclusions regarding structural transitions were based on studies from the material that was recovered after applying the shock drive or dynamic measurement of macroscopic quantities, such as density and pressure. As a consequence, there is a speculation in understanding the dynamic processes for shock synthesis of diamond and lonsdaleite^20^,^21^,^22^,^23^,^24^,^25^,^26^,^27^ and conclusions regarding meteor impacts^2^,^5^. Particularly, the different concepts involve nucleation^24^, plane-sliding and buckling^22^, wave-buckling and slipping^27^ or the intermediate formation of so-called layered diamond^20^, which is a mixture of hexagonal and cubic diamond^23^. These models give very different results under which conditions, or if at all, diamond or lonsdaleite are formed from graphite. Moreover, while a fast martensitic process on nanosecond timescale has been accepted for compressing highly oriented graphite samples along the *c* axis^4^,^17^, much slower diffusion-based mechanisms are thought to be more effective for highly defective graphite samples^28^.

In the following, we address these questions in an unprecedented way by applying ultrafast X-ray diffraction for *in situ* structure measurements of the shock-induced transition from graphite to diamond. Our results show nanosecond formation of the diamond structure after shock compressing pyrolytic as well as porous polycrystalline graphite samples. At the highest pressures, we find evidence for a relatively pure lonsdaleite structure when compressing pyrolytic graphite, whereas the porous polycrystalline samples are transferred to a complex liquid in this regime.

## Results

### Experimental method

**Figure 1:**
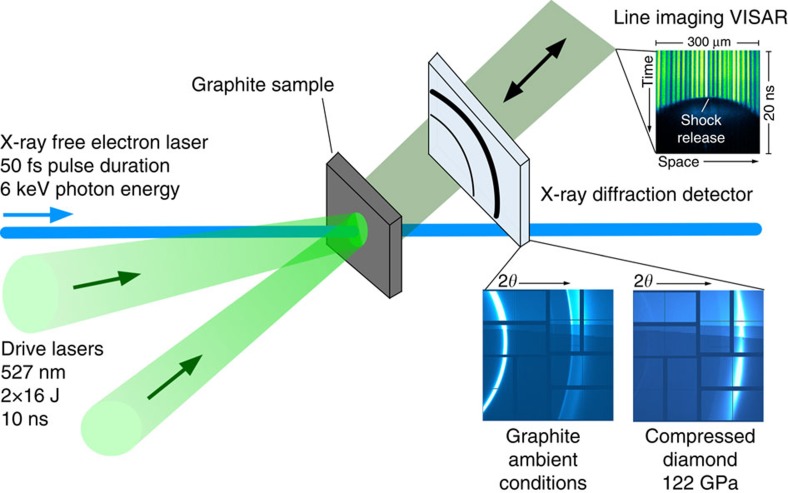
Schematic of the experimental setup at the Matter at Extreme Conditions endstation of the Linac Coherent Light Source. Two high-energy laser beams drive shock waves into graphite samples generating pressures from 20 to 230 GPa. The shock transit times of few nanoseconds are recorded by a VISAR system, which detects the shock-induced reflectivity drop of a 100-nm thick aluminum coating when the shock exits on the target rear side. The microscopic state is probed by a single X-ray pulse with 6 keV photon energy and 50 fs pulse duration. X-ray diffraction is recorded by a large area X-ray detector.

The experiments reported here were performed at the Matter at Extreme Conditions endstation of the Linac Coherent Light Source of Stanford National Accelerator Laboratory^29^. Here, the newly available combination of high-energy lasers (≳10 J per pulse) with an X-ray free electron laser allows for femtosecond X-ray diffraction to directly access the kinetics of the shock-induced transition. A schematic of the experiment is shown in Fig. 1.

The recorded diffraction angle *θ* is directly connected to the lattice spacing *d* via the Bragg relation


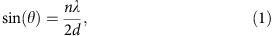


where *n* is the diffraction order, and *λ* the wavelength of the incident light source. For the symmetric cubic diamond crystal structure, the lattice spacing given by the (111) diffraction peak directly determines the density *ρ*∝*d*^−3^. Knowing the density of the shocked state *ρ*_1_ as well as the shock transit time, and thus the shock velocity *v*_S_, allows for inferring the pressure *p* using the Rankine–Hugoniot relations, which simply apply mass, momentum and energy conservation^30^


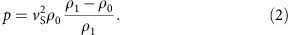


Here, *ρ*_0_ is the initial density of the graphite sample.

### Pyrolytic graphite

**Figure 2:**
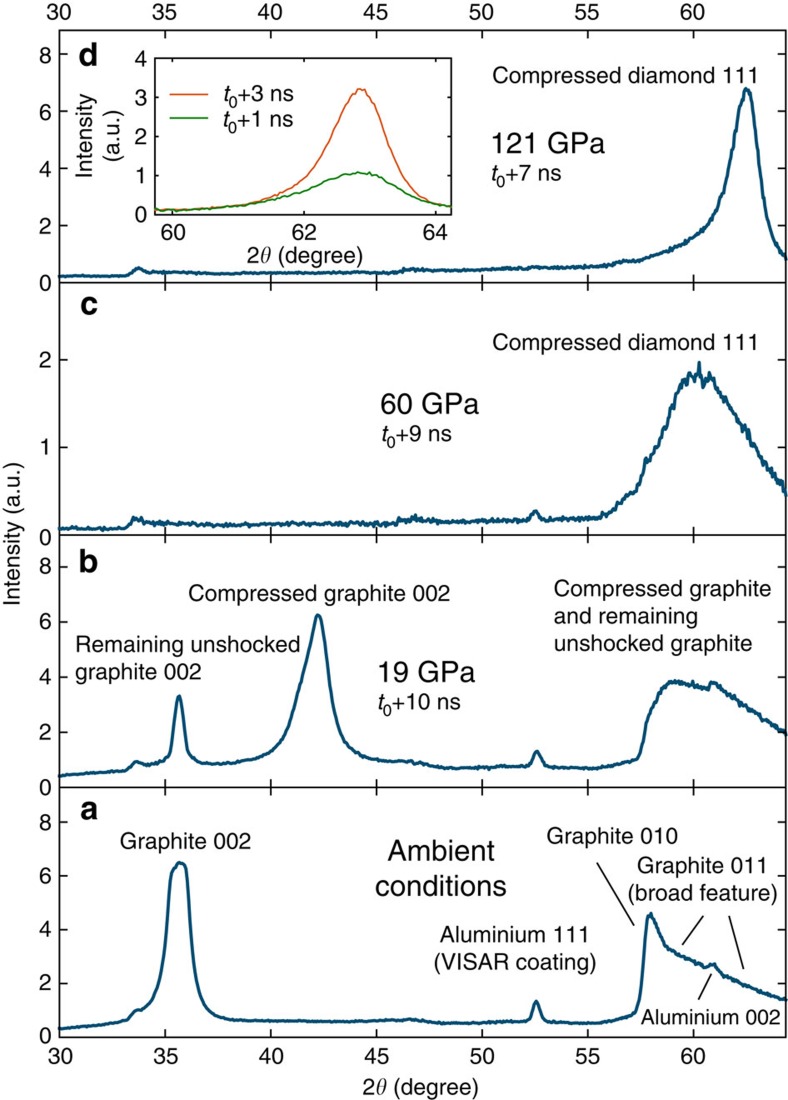
Transition from pyrolytic graphite to diamond. X-ray diffraction data of (**a**) cold and (**b**–**d**) compressed pyrolytic graphite samples, driven parallel to the graphite *c* axis; *t*_0_ is denoting the start of the drive laser pulse. For 19 GPa, diffraction shows graphite, which is mainly compressed along the *c* axis, together with some cold material from the rear side of the sample not reached by the shock. For 60 GPa at exactly the moment when VISAR records the shock having traversed the whole sample, every signature from graphite vanishes. In this case, only a broad cubic diamond (111) diffraction peak remains. For higher pressures (for example, 122 GPa), the width of the peak decreases due to the reduced transition time and we observe the formation of a sharp Bragg reflection within 1 ns.

**Figure 3:**
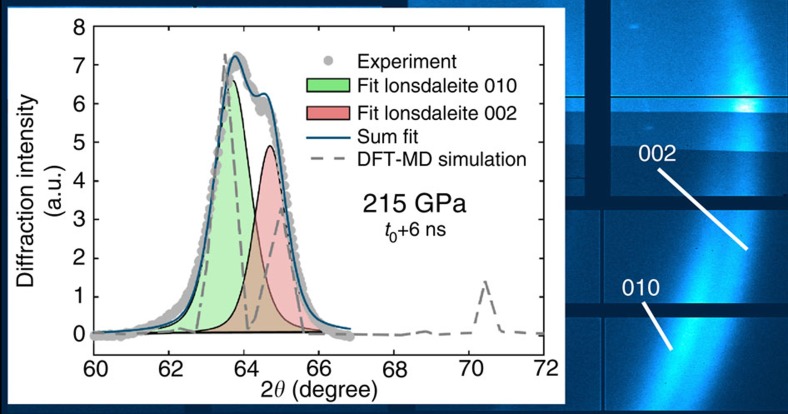
Diffraction from lonsdaleite. When compressing pyrolytic graphite to pressures above 170 GPa, we observe two strong diffraction peaks consistent with lonsdaleite (010) and (002) planes. The grey-dashed line shows a DFT-MD simulation of the lonsdaleite phase at 4.4 g cm^−3^ and 6,000 K, resulting in a pressure of 205 GPa, which is in qualitative agreement with the observed diffraction pattern. As the range of detectable diffraction angles ends at 2*θ*=68 degrees in our experiments, the (011) peak at 2*θ*=70.5 degrees is not recorded. The occurrence of partial diffraction rings shows a preferred orientation of the crystallites that is compatible with the preferred orientation of the initial pyrolytic graphite sample.

Figure 2 shows X-ray diffraction results for oriented pyrolytic graphite (*ρ*_0_=2.21 g cm^−3^) driven parallel to the graphite *c* axis with different drive pressures. For ∼20 GPa, we observe the (002) Bragg reflection shifting to larger diffraction angles, thus providing a signature of compressed graphite. For pressures above ∼55 GPa, the graphite (002) signature has completely vanished, whereas a broad peak at ∼60 degrees appears. This new feature is in agreement with the (111) Bragg reflection of diamond. The large width of the diffraction peak at this pressure most probably documents remaining disorder (that is, very small diamond crystallites) and thus implies that the transition from graphite to diamond is not fully completed within the shock transit time of ∼9 ns. Higher drive pressures result in faster transition times, reducing the width of the (111) diamond diffraction peak due to less disorder, that is, creation of larger diamond crystallites. For drive pressures above 100 GPa, we observe the formation of diamond on timescales less than 1 ns (inset in Fig. 2d). For these pyrolytic samples at shock pressures higher than 170 GPa, we see two diffraction ring fragments emerging that are clearly caused by different crystal planes showing some degree of texture (see Fig. 3), conserving the preferred crystallite orientation of the pyrolytic samples (see Supplementary Figs 1 and 2). The inferred spacing is compatible with diffraction from the (010) and (002) planes of lonsdaleite. For this structure, we obtain an average *c*/*a* lattice constant ratio of ∼1.69, which is slightly above the ideal value of 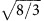 for a wurtzite lattice with equidistant atoms. This reasonably follows the trend given by reported values for ambient conditions^8^ (*c*/*a*=1.64) and simulations for 20 GPa (ref. 23; *c*/*a*=1.66). Density functional theory coupled with molecular dynamics (DFT-MD) simulations of the lonsdaleite structure at 4.4 g cm^−3^ and 6,000 K, resulting in a pressure of 205 GPa, show a very good agreement with our diffraction measurements.

The observed diffraction pattern is only compatible with lonsdaleite and not with any other structure that has been predicted for this pressure regime. Faulted and twinned cubic diamond, as shown by Nemeth *et al.*^2^, would result in a cubic diamond main peak with small side peaks due to the defects. Furthermore, a mix of cubic and hexagonal diamond, the so-called layered diamond struture, would not result in the observed clear doublet of peaks^20^. Other more complicated polymorphs show many more diffraction maxima^23^ and can certainly be excluded by our measurements. Hexagonal lonsdaleite being favoured at higher drive pressures is conceivable since the direct transition path to cubic diamond requires more lattice distortions^22^,^24^,^26^. Recent MD calculations^27^ demonstrate that plane sliding of the graphite basal planes to rhombohedral graphite, which in turn would lead to the formation of diamond, is prevented by the emergence of few interplanar sp^3^ bonds, when the spacing between the graphite basal planes falls below a certain distance. For this case, these simulations predict the formation of lonsdaleite^27^. Our experiments indicate that this limit is reached by dynamic shock compression of pyrolytic graphite above 170 GPa.

**Figure 4:**
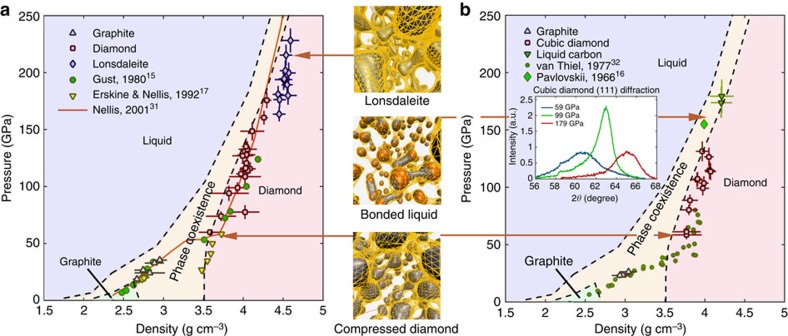
Summary of the experimental results. (**a**) Recorded pressure–density diagram for compressed pyrolytic graphite (*ρ*_0_=2.21 g cm^−3^) compared with literature data without structure information and a first principles phase diagram^33^. At lower pressures, there is very good agreement, whereas at higher pressures, due to the formation of lonsdaleite, we observe higher densities than predicted by a proposed shock Hugoniot^31^, which instead suggests a transition to the liquid. (**b**) Recorded pressure–density diagram for porous polycrystalline graphite (*ρ*_0_=1.84 g cm^−3^) compared with experiments without structure information^16^,^32^ and a first principles phase diagram. Comparable to the pyrolytic samples, the diamond formation is not fully completed within few nanoseconds at lower pressures, resulting in a broad peak which sharpens up to ∼100 GPa. At higher pressures, however, the increasing temperature leads to melting of the diamond structure, resulting in broader and fainter diffraction peaks in agreement with a bonded liquid^18^. No signature of lonsdaleite is observed when compressing porous graphite.

Figure 4 combines the recorded microscopic crystal structure with the macroscopic quantities density and pressure. Applying the simple relation between Bragg angle and lattice spacing, and thus density, in combination with equation (2), we can create an experimental pressure–density diagram, giving a line of states achievable by shock compression, the shock Hugoniot curve (all experimental values including error estimates can be found in Supplementary Tables 1 and 2). Classical Hugoniot measurements usually determine only macroscopic quantities like density and pressure via velocity measurements. As such, the shock Hugoniot is one of few experimentally accessible quantities capable of benchmarking high-pressure equation of state theories, from which, for example, the temperature of the created states can be deduced. Only based on these models, it is then possible to draw conclusions on microscopic properties. In contrast, our measurements directly record the microscopic structure of the sample in addition to determining density and pressure. Thus, they give a more complete picture joining both microscopic and macroscopic observations to an absolute equation of state measurement.

For compressed pyrolytic graphite, our results connect and overlap with established literature data at lower pressures^15^,^16^,^17^,^31^,^32^ as well as first principles simulations of the carbon phase diagram^33^ (see Fig. 4a). At higher pressures, our data extend to a regime which has not been well characterized before. For states which remain in the graphite phase, it needs to be considered that graphite mainly compresses along the *c* axis of the unit cell^34^. The (002) diffraction peak position directly determines the lattice spacing *d*_*c*_ along the *c* axis and using the relation 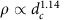 from static compression experiments^35^, we can infer the density of the compressed graphite states. The rearrangement in the microscopic structure, that is, the graphite–diamond transition, is clearly visible due to a density jump at the transition pressure of ∼50 GPa and a lower compressibility of the diamond states. We do not observe a two-wave structure as, for example, Erskine and Nellis^17^ did for this transition after shock compression of pyrolytic graphite samples with a higher grade of orientation. These experiments achieved the transition already at 27 GPa, while the two-wave structure was overdriven at 50 GPa. In agreement with our experiments, Erskine and Nellis did not record a two-wave structure for graphite samples with lower grade of orientation. At higher pressures, we observe a second smaller jump in density at ∼170 GPa, pointing at the transition to lonsdaleite consistent with a slightly increased energy that is required for the structural transition compared with diamond^36^. In turn, we obtain higher densities than predicted by a proposed shock Hugoniot^31^ that does not take the formation of lonsdaleite into account in this regime. However, this phase exhibits even higher stiffness, supporting predictions that the hardness of cubic diamond is exceeded by its hexagonal polymorph^9^ and possibly leading to a re-connection with the proposed Hugoniot line in the liquid phase. Using a very recent multi-phase equation of state model for carbon^37^, we expect the temperatures of the states, which remain in the graphite phase in the order of 4,000 K, 4,000–6,000 K for the diamond data and 6,000–7,000 K for the measurements, which show the lonsdaleite structure.

### Porous polycrystalline graphite

For porous polycrystalline samples, we obtain slightly different results (see Fig. 4b). When compressing porous samples, the voids close instantly nearly without resistance, resulting in an entropy increase. Further compression then leads to states of higher entropy and thus temperature compared with shock compression of the higher density pyrolytic samples to the same pressure^30^. For porous polycrystalline graphite, we record nanosecond formation of diamond with a transition threshold at slightly lower pressure compared with the pyrolytic samples. The width of the diffraction peak is marginally larger than for pyrolytic samples. This indicates that only slightly smaller diamond crystallites are formed for the polycrystalline samples. Starting from ∼100 GPa, the diffraction again broadens and weakens due to melting and, instead of reaching the hexagonal phase, a bonded liquid is formed, which is in good agreement with the first principles phase diagram. It has been shown that short-time bonding remains active in liquid carbon, probably up to temperatures of more than 20,000 K (ref. 38). As the liquid structure is diamond-like, the diffraction signatures do not immediately vanish or shift above the melting line. Again using a recent multi-phase equation of state model^37^, we expect the temperatures of the states which remain in the graphite phase in the order of 4,000–5,000 K, 5,000–8,000 K for the diamond data and temperatures exceeding 8,000 K for the data points, which show the transition to the liquid.

## Discussion

The data of dynamic diamond formation presented here resolve controversies on the shock-induced graphite to diamond transition: a fast transition from graphite to diamond on nanosecond timescale does not require highly oriented graphite samples and shock loading perpendicular to the *c* axis. Furthermore, recording the first clear diffraction signal of the meta-stable hexagonal diamond polymorph, that is not obscured by dominant cubic diamond or graphite, demonstrates that this state exists. Finally, observing lonsdaleite only for pyrolytic graphite samples compressed along the *c* axis to very high pressures suggests that sliding of the graphite basal planes and thus diamond formation can be prevented under these conditions. Hence, the picture that lonsdaleite synthesis is highly difficult applying static pressure but can be formed in violent impact events is strongly supported.

## Methods

### Laser-driven shock compression

The graphite samples (pyrolytic graphite, GoodFellow C000720, *ρ*_0_=2.21 g cm^−3^, mosaicity ∼10 degrees and porous polycrystalline graphite, SGL Carbon R6650, *ρ*_0_=1.84 g cm^−3^, 10% volume porosity, 5 μm average grain size) were compressed using two pulsed high-energy lasers (527 nm, up to 16 J per beam, 10 ns pulse duration, 150–200 μm focal spot diameter, smoothed with random phase plates). The samples were laser-cut out of larger graphite pieces and then polished to achieve a surface roughness of 0.1 μm. The sample thickness was varied between 60 and 100 μm and had individually been characterized for each sample with an accuracy of ∼0.5 μm. The shock transit time was recorded with a VISAR system, which monitored the drop in reflectivity of a 100 nm aluminium coating on the sample rear side at the moment of shock release. The VISAR system at MEC has two streak cameras and the transit time was determined as the average of the two cameras. The arrival time of the drive laser was determined by the same streak cameras as used for the VISAR system by scattering light of the drive laser beams from the exact position of sample interaction into the VISAR system. Typical transit times varied between 6 and 12 ns, depending on sample thickness and drive pressure. Recording transit times for different sample thickness and using the spatial resolution of the VISAR, sufficient shock steadiness as well as shock planarity could be guaranteed for the region that was irradiated by the X-ray beam. This was confirmed by measurements using graphite samples with a LiF window on the rear side, where a very steady interface velocity could be monitored. Using this interface velocity and the shock velocity in graphite, as well as assuming the LiF equation of state to be well known, density and pressure in the shocked graphite sample can be inferred in principle. These results are in agreement with the X-ray diffraction method, but by far not as accurate for our experiments (particularly in terms of density) and cannot provide additional insights.

### Ultrafast X-ray diffraction

The graphite samples were probed using the LCLS XFEL beam in SASE mode (5.9–6.1 keV photon energy, 0.3% spectral bandwidth, 50 fs pulse duration, 20 μm spot size, ∼3 mJ per pulse corresponding to 3 × 10^12^ photons per pulse). The input spectrum was recorded for each run with a high-resolution silicon crystal spectrometer. The timing of the X-ray pulse relative to the drive laser was chosen to probe the sample slightly before or exactly at the moment when the shock wave had traversed the whole sample. X-ray diffraction was measured by an 8 × 8 cm^2^ Cornell-Stanford Pixel Array detector (CSPAD) at 11 cm distance to the sample covering diffraction angles from 27 to 68 degrees on top of the XFEL beam axis. In this way, interfering effects due to the horizontal polarization of the X-ray beam could be avoided. Angular calibration for the recorded 2*θ* range of 20–68 degrees was obtained by diffraction of a LaB_6_ powder sample.

## Additional information

**How to cite this article:** Kraus, D. *et al.* Nanosecond formation of diamond and lonsdaleite by shock compression of graphite. *Nat. Commun.* 7:10970 doi: 10.1038/ncomms10970 (2016).

## Supplementary Material

Supplementary Information Supplementary figures 1-2 and Supplementary Tables 1-2.

## References

[b1] DeCarli P. S. & Jamieson J. C. Formation of diamond by explosive shock. Science 133, 1821–1822 (1961). 17818997 10.1126/science.133.3467.1821

[b2] Nemeth P. *et al.* Lonsdaleite is faulted and twinned cubic diamond and does not exist as a discrete material. Nat. Commun. 5, 5447 (2014). 25410324 10.1038/ncomms6447

[b3] Bovenkerk H. P., Bundy F. P., Hall H. T., Strong H. M. & Wentorf R. H. Preparation of diamond. Nature 184, 1094–1098 (1959).

[b4] Erskine D. J. & Nellis W. J. Shock-induced martensitic phase transformation of oriented graphite to diamond. Nature 349, 317–319 (1991).

[b5] Hanneman R. E., Strong H. M. & Bundy F. P. Hexagonal diamonds in meteorites: implications. Science 166, 995–997 (1967). 17830485 10.1126/science.155.3765.995

[b6] Ross M. The ice layer in Uranus and Neptune-diamonds in the sky? Nature 292, 435–436 (1981).

[b7] Bundy F. P. & Casper J. S. Hexagonal diamond-a new form of carbon. J. Chem. Phys. 46, 3437 (1967).

[b8] Frondel C. & Marvin U. B. Lonsdaleite, a hexagonal polymorph of diamond. Nature 217, 587–589 (1967).

[b9] Pan Z., Sun H., Lang Y. & Chen C. Harder than diamond: superior indentation strength of wurtzite BN and lonsdaleite. Phys. Rev. Lett. 102, 055503 (2009). 19257519 10.1103/PhysRevLett.102.055503

[b10] Kvasnytsya V. *et al.* New evidence of meteoritic origin of the Tunguska cosmic body. Planet. Space Sci. 84, 131–140 (2013).

[b11] Hough R. M. *et al.* Diamond and silicon carbide in impact melt rock from the Ries impact crater. Nature 378, 41–44 (1995).

[b12] Kennet D. J. *et al.* Shock-synthesized hexagonal diamonds in Younger Dryas boundary sediments. Proc. Natl Acad. Sci. USA 106, 12623–12628 (2009). 19620728 10.1073/pnas.0906374106 PMC2722287

[b13] Carlisle D. B. & Braman D. R. Nanometre-size diamonds in the Cretaceous/Tertiary boundary clay of Alberta. Nature 352, 708–709 (1991).

[b14] Salzmann C. G., Murray B. J. & Shephard J. J. Extent of stacking disorder in diamond. Diamond Relat. Mater. 59, 69–72 (2015).

[b15] Gust W. H. Phase transition and shock-compression parameters to 120 GPa for three types of graphite and for amorphous carbon. Phys. Rev. B 22, 4744–4756 (1980).

[b16] Pavlovskii B. J. & Drakin V. P. Concerning the metallic phase of carbon. JETP Lett. 4, 116 (1966).

[b17] Erskine D. J. & Nellis W. J. Shock-induced martensitic phase transformation of highly oriented graphite to diamond. J. Appl. Phys. 71, 4882–4886 (1992).

[b18] Kraus D. *et al.* Probing the complex ion structure in liquid carbon at 100 GPa. Phys. Rev. Lett. 111, 255501 (2013). 24483747 10.1103/PhysRevLett.111.255501

[b19] Ohtani E. *et al.* Formation of high-pressure minerals in shocked L6 chondrite Yamato 791384: constraints on shock conditions and parent body size. Earth Planet. Sci. Lett. 227, 505–515 (2004).

[b20] Mundy C. J. *et al.* Ultrafast transformation of graphite to diamond: an *ab initio* study of graphite under shock compression. J. Chem. Phys. 128, 184701 (2008). 18532830 10.1063/1.2913201

[b21] Winey J. M. & Gupta J. M. Shock-compressed graphite to diamond transformation on nanosecond time scales. Phys. Rev. B 87, 174104 (2013).

[b22] Scandolo S., Bernasconi M., Chiarotti G. L., Focher P. & Tosatti E. Pressure-induced transformation path of graphite to diamond. Phys. Rev. Lett. 74, 4015–4018 (1995). 10058391 10.1103/PhysRevLett.74.4015

[b23] Ribeiro F. J., Tangney P., Louie S. G. & Cohen M. L. Structural and electronic properties of carbon in hybrid diamond-graphite structures. Phys. Rev. B 72, 214109 (2005).

[b24] Khaliullin R. Z., Eshet H., Kühne T. D., Behler J. & Parrinello M. Nucleation mechanism for the direct graphite-to-diamond phase transition. Nat. Mater. 10, 693–697 (2011). 21785417 10.1038/nmat3078

[b25] Kurdyumov A. V., Britun V. F., Yarosh V. V., Danilenko A. I. & Zelyavskii V. B. The influence of the shock compression conditions on the graphite transformations into lonsdaleite and diamond. J. Superhard Mater. 34, 19–27 (2012).

[b26] Pineau N. Molecular dynamics simulations of shock compressed graphite. J. Phys. Chem. C 117, 12778–12786 (2013).

[b27] Xie H., Yin F., Yu T., Wang J.-T. & Liang C. Mechanism for direct graphite-to-diamond phase transition. Sci. Rep. 4, 5930 (2014). 25088720 10.1038/srep05930 PMC4120013

[b28] Hirai H., Kukino S. & Kondo K. Predominant parameters in the shock induced transition from graphite to diamond. J. Appl. Phys. 78, 3052–3059 (1995).

[b29] Gauthier M. *et al.* New experimental platform to study high density laser-compressed matter. Rev. Sci. Instrum. 85, 1E616 (2014). 10.1063/1.4896175 25430362

[b30] Zeldovic Y. B. & Raizer Y. P. Physics of Shock Waves and High-Temperature Hydrodynamic Phenomena Academic Press, New York and London (1966).

[b31] Nellis W. J., Mitchell A. C. & McMahan A. K. Carbon at pressures in the range 0.1-1 TPa (10 Mbar). J. Appl. Phys. 90, 696–698 (2001).

[b32] van Thiel M. Compendium of shock wave data. *Lawrence Livermore Laboratory Report* UCRL-50108 (1977).

[b33] Wang X., Scandolo S. & Car R. Carbon phase diagram from *ab initio* molecular dynamics. Phys. Rev. Lett. 95, 185701 (2005). 16383918 10.1103/PhysRevLett.95.185701

[b34] Nakayama A. *et al.* Compression of polyhedral graphite up to 43 GPa and x-ray diffraction study on elasticity and stability of the graphite phase. Appl. Phys. Lett. 84, 5112–5114 (2004).

[b35] Zhao Y. X. & Spain I. L. X-ray diffraction data for graphite to 20 GPa. Phys. Rev. B 40, 993–997 (1989). 10.1103/physrevb.40.993 9991920

[b36] Wu B. R. & Xu J. Total energy calculations of the lattice properties of cubic and hexagonal diamond. Phys. Rev. B 57, 13355–13358 (1998).

[b37] Benedict L. X. *et al.* Multiphase equation of state for carbon addressing high pressures and temperatures. Phys. Rev. B 89, 224109 (2014).

[b38] Kraus D. *et al.* The complex ion structure of warm dense carbon measured by spectrally resolved x-ray scattering. Phys. Plasmas 22, 056307 (2015).

