# Supplementary material for: Nanosecond formation of diamond and lonsdaleite by shock compression of graphite
Source: Nat Commun. 2016 Mar 14;7:10970. doi: 10.1038/ncomms10970 (PMC4793081; doi:10.1038/ncomms10970)
Supplement: Supplementary Information — Supplementary figures 1-2 and Supplementary Tables 1-2. [file ncomms10970-s1.pdf]

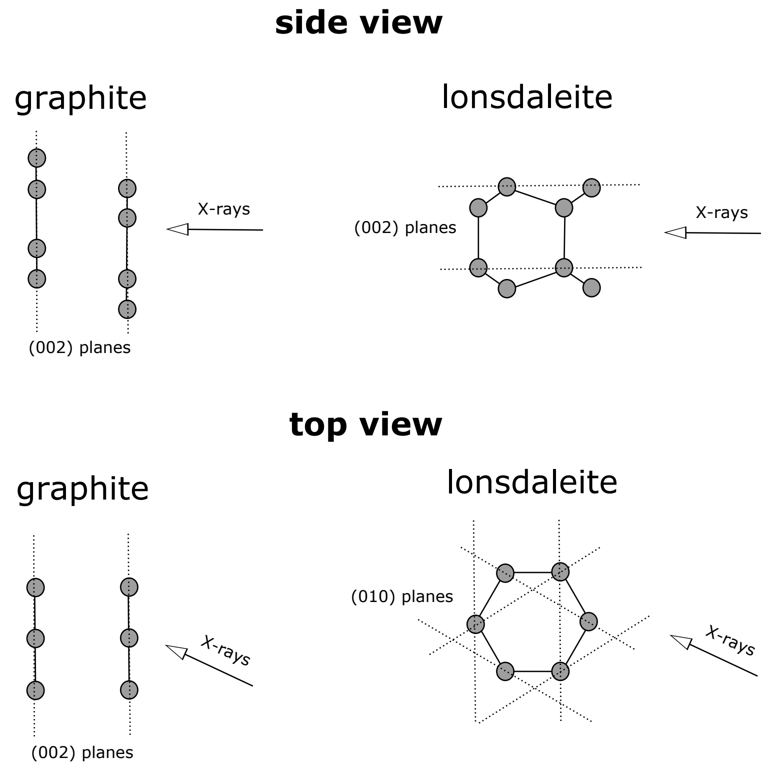

Supplementary Figure 1. Preferred crystallite orientation in the pyrolytic graphite samples and subsequent preferred orientation of the lonsdaleite crystallites following the predicted transition path from graphite to lonsdaleite. Shock compression is applied perpendicular to the graphite (002) planes while the X-rays penetrate the graphite samples with a 24 degrees angle of incidence in the horizontal plane.

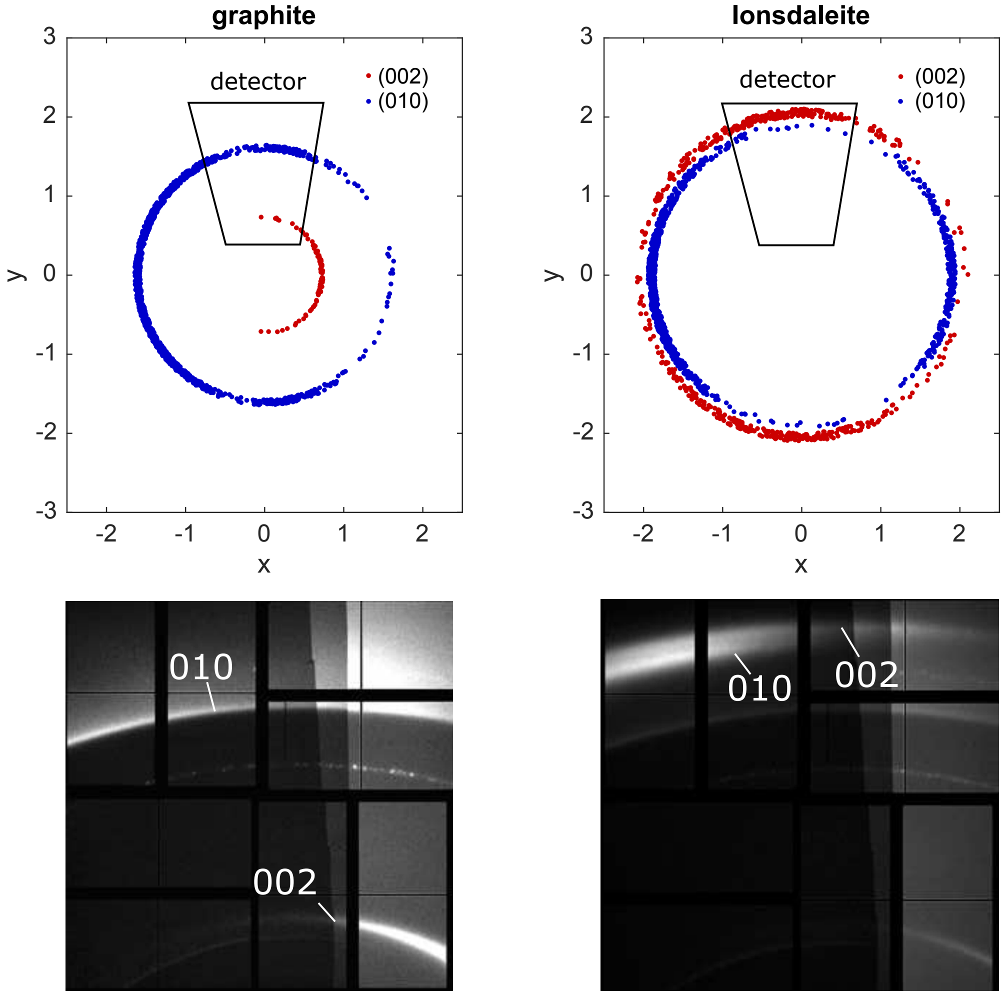

Supplementary Figure 2. Simple ray-tracing calculations for the preferred crystallite orientations assuming a Gaussian distribution for the angular deviations with 20 degrees FWHM using the (002) and (010) reflection for lonsdaleite. Qualitatively, this is in very good agreement with the observed texture on the diffraction detector for both pyrolytic graphite and lonsdaleite. For graphite, the (002) planes are preferentially oriented perpendicular to the horizontal plane and result in preferred reflection of the X-rays to the side where the Bragg condition is more likely to be met due to the 24 degrees angle of incidence of the X-rays in the horizontal plane. For lonsdaleite, the resulting preferred orientation of the (002) planes is horizontal, giving a preferential reflection of the X-rays in upward or downward direction. The lonsdaleite (010) planes are all preferentially oriented perpendicular to the horizontal plane and thus primarily reflect the X-rays to the left and right in this geometry.

Supplementary Table 1. Results for pyrolytic graphite.

v_s [km s^{-1}]	Δv_s [km s^{-1}]	ρ_1 [g cm^{-3}]	$\Delta\rho_1$ [g cm^{-3}]	p [GPa]	Δp [GPa]	structure of shocked state
6.83	0.07	2.73	0.09	20.0	0.8	graphite
6.95	0.14	2.67	0.05	18.6	0.8	graphite
7.03	0.07	2.83	0.19	24.1	1.7	graphite
7.80	0.09	2.75	0.11	26.6	1.2	graphite
7.92	0.10	2.89	0.12	32.8	1.6	graphite
7.94	0.09	2.95	0.09	35.4	1.4	graphite
8.40	0.11	3.58	0.14	59.8	2.8	diamond
8.82	0.29	4.02	0.18	77.6	6.1	diamond
9.06	0.12	3.72	0.19	73.9	4.3	diamond
10.04	0.15	3.82	0.25	94.1	6.8	diamond
10.11	0.15	3.93	0.15	98.9	4.8	diamond
10.44	0.16	4.02	0.20	108.6	6.3	diamond
10.64	0.17	3.97	0.06	111.0	3.8	diamond
10.64	0.17	4.06	0.06	114.1	4.1	diamond
10.67	0.17	4.09	0.07	115.5	4.2	diamond
10.74	0.18	4.02	0.07	114.8	4.4	diamond
10.79	0.17	4.03	0.07	116.2	4.1	diamond
10.97	0.18	4.04	0.06	120.5	4.4	diamond
11.29	0.19	4.04	0.11	127.8	5.5	diamond
11.33	0.19	3.99	0.13	126.9	6.0	diamond
11.49	0.23	4.05	0.11	132.7	6.5	diamond
11.62	0.19	4.03	0.05	135.1	4.8	diamond
12.05	0.24	4.11	0.15	148.5	8.1	diamond
12.13	0.21	4.45	0.08	163.5	6.4	lonsdaleite
12.28	0.22	4.26	0.05	160.4	6.1	diamond
12.57	0.24	4.56	0.11	179.9	8.1	lonsdaleite
12.58	0.23	4.46	0.08	176.2	7.2	lonsdaleite
12.77	0.24	4.44	0.07	180.8	7.5	lonsdaleite
12.80	0.24	4.29	0.06	175.7	6.9	diamond
12.91	0.24	4.55	0.07	189.3	7.6	lonsdaleite
13.12	0.27	4.51	0.11	193.8	9.2	lonsdaleite
13.13	0.25	4.51	0.07	194.5	7.9	lonsdaleite
13.23	0.26	4.57	0.08	199.7	8.6	lonsdaleite
13.35	0.28	4.53	0.07	201.7	9.1	lonsdaleite
13.79	0.27	4.54	0.08	215.3	9.3	lonsdaleite
14.11	0.28	4.59	0.11	228.2	10.6	lonsdaleite

Supplementary Table 2. Results for polycrystalline porous graphite.

v_s [km s^{-1}]	Δv_s [km s^{-1}]	ρ_1 [g cm^{-3}]	$\Delta\rho_1$ [g cm^{-3}]	p [GPa]	Δp [GPa]	structure of shocked state
5.80	0.29	2.97	0.08	23.5	2.4	graphite
5.81	0.30	2.93	0.10	23.1	2.5	graphite
6.03	0.37	3.04	0.11	26.3	3.4	graphite
7.88	0.16	3.77	0.19	58.5	3.8	diamond
8.06	0.28	3.77	0.18	61.2	5.2	diamond
9.19	0.22	3.80	0.11	80.3	4.5	diamond
9.65	0.24	3.81	0.10	88.6	5.0	diamond
10.01	0.29	3.97	0.08	99.1	6.2	diamond
10.22	0.29	3.99	0.06	103.6	6.1	diamond
10.46	0.30	3.90	0.07	106.6	6.4	diamond
10.49	0.28	3.91	0.06	107.2	6.0	diamond
10.61	0.34	4.07	0.07	113.7	7.5	diamond
10.68	0.34	4.06	0.06	114.8	7.6	diamond
11.22	0.35	4.05	0.06	126.5	8.1	diamond
11.53	0.36	3.97	0.08	131.3	8.6	diamond
12.95	0.45	4.21	0.13	173.7	13.3	diamond/liquid carbon
13.15	0.49	4.21	0.15	179.4	14.7	diamond/liquid carbon
